# One‐way valves in breathing tubing reduce dead space during spontaneous breathing in anesthetized piglets

**DOI:** 10.1002/pdi3.2502

**Published:** 2024-08-04

**Authors:** Pan Li, Weiping Wang, Wen Gao, Yanling Tan, Yu Hu, Li Jiang

**Affiliations:** ^1^ Department of Anesthesiology, Children's Hospital of Chongqing Medical University National Clinical Research Center for Child Health and Disorders Ministry of Education Key Laboratory of Child Development and Disorders Chongqing Key Laboratory of Child Neurodevelopment and Cognitive Disorders Chongqing China; ^2^ Department of Neurology, Children's Hospital of Chongqing Medical University National Clinical Research Center for Child Health and Disorders Ministry of Education Key Laboratory of Child Development and Disorders Chongqing Key Laboratory of Child Neurodevelopment and Cognitive Disorders Chongqing China

**Keywords:** breathing tubing, circle breathing system, dead space, spontaneous breathing

## Abstract

The circle breathing system was unsafe for spontaneous breathing because of hypercapnia during anesthesia. Few studies have examined the minimizing dead space in breathing tubing. This study investigated one‐way valves in the breathing tubing during spontaneous breathing in piglets. Six female piglets aged 68–71 days spontaneously breathed sevoflurane for 4 h randomly via traditional or anti‐rebreathing tubing. Arterial carbon dioxide tension (paCO_2_) and respiratory characteristics were used to assess spontaneous breathing efficiency. mRNA‐based methods, immunohistochemistry, and histology were used to assess the lungs. After induction, all piglets had mild hypercapnia. Those who breathed via traditional tubing experienced severe hypercapnia and required assisted ventilation (mean [95% confidence interval for mean]: 3 [0.5; 5.5] times) over 4 h. Piglets who breathed via anti‐rebreathing tubing were able to normalize without assisted ventilation in less than 3 h and maintained. paCO_2_ was higher in the traditional group than the anti‐rebreathing group at 3 and 4 hours (46.3 [42.1; 50.5] vs. 38.3 [34.1; 42.5] mmHg, *p* = 0.020; 46.3 [42.6; 50.0] vs. 40.7 [37.0; 44.4] mmHg, *p* = 0.040). However, one‐way valves increased resistance to breathing. For the lungs, mRNA‐based methods indicated higher expressions of cyclin‐dependent kinase, cell division cycle 20, and cyclin B2 in the traditional group; immunohistochemistry identified higher expression of phosphorylated histone 2AX in the traditional group; histology showed similar damage between the groups. These findings suggest that one‐way valves inside breathing tubing reduced dead space during spontaneous breathing and enhanced inhalation anesthesia advantages in the circle breathing system.

## Graphical abstract

One‐way valves inside the Y‐piece of breathing tubing reduce dead space during spontaneous breathing and enhance inhalation anesthesia advantages in the circle breathing system. Breathing tubing with one‐way valves can make sevoflurane sedation efficient and safe during spontaneous breathing.

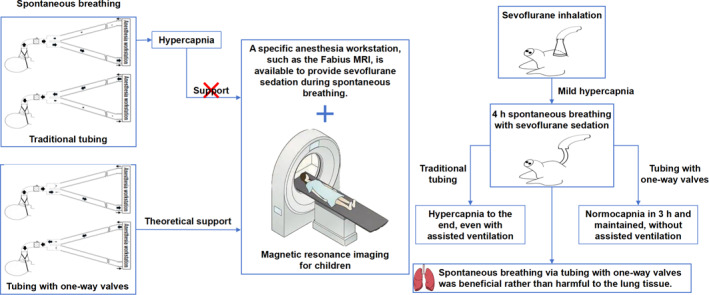

## 1 INTRODUCTION

**FIGURE 1:**
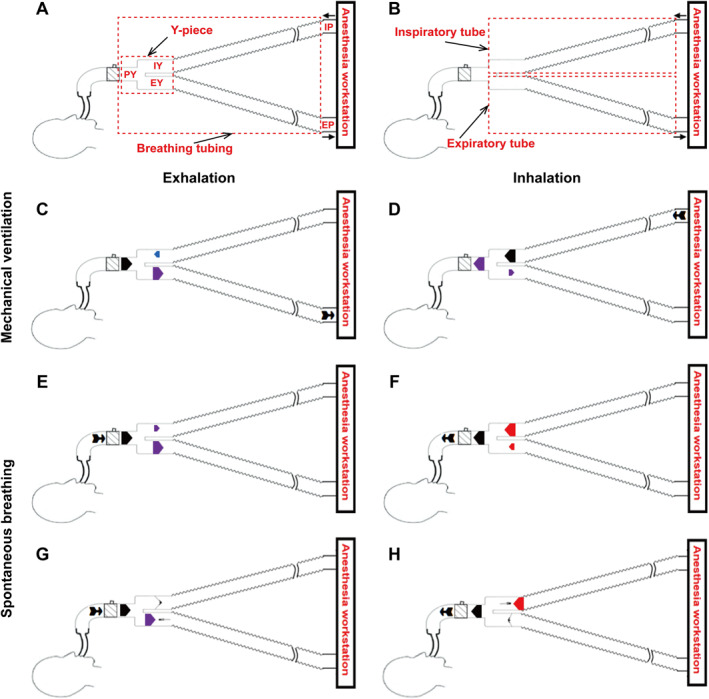
Diagrams of the circle breathing system and gas flow pattern at the beginning of breathing. (A, B) Diagrams of the circle breathing system. (C, D) Mechanical ventilation via traditional tubing. (E, F) Spontaneous breathing via traditional tubing. (G, H) Spontaneous breathing via anti‐rebreathing tubing. In (C, D, E, F, G, H), the thick arrows indicate the gas flow direction and flow rate in different parts of the Y‐piece; the black arrow indicates the specific location at which the main force is propelling the gas flow within the Y‐piece; the red arrow indicates a specific location in the Y‐piece at which there is a higher pressure value than the main force; the blue arrow indicates a specific location in the Y‐piece at which there is an almost equal pressure value to the main force; the purple arrow indicates a specific location in the Y‐piece at which there is a lower pressure value than the main force. ➽ in (C) indicates the specific location of breathing tubing nearest the expiratory port of the anesthesia workstation (although the elastic properties of the lung and chest wall are the main force propelling the gas flow during the exhalation stage in mechanical ventilation, this stage is activated by the opening of the one‐way valve in the expiratory port); and ➽ in (D, E, F, G, H) indicate the specific location at which the main force is propelling the gas flow within the breathing tubing. EP, expiratory port of the anesthesia workstation; EY, expiratory Y‐piece; IP, inspiratory port of the anesthesia workstation; IY, inspiratory Y‐piece; PY, patient Y‐piece.

The circle breathing system (Figure 1A,B) is the most commonly used for the gas mixture flows between the anesthesia workstation and patient. Its main advantages include carbon dioxide (CO_2_) elimination, anesthetic gas savings, and operating room pollution control.^1^ Nevertheless, due to inadequate gas exchange, the patient's spontaneous breathing via the circle breathing system ran the risk of hypercapnia.^2^ CO_2_ is a powerful stimulus to the respiratory control system, as even a slight increase in CO_2_ level can result in increasing both respiratory frequency and tidal volume (TV),^3^ which can seriously disturb clinical procedures. Hypercapnia has negative impacts on various organs throughout the body, including the lungs^4^ and brain.^5^

In the circle breathing system, when it changes from mechanical ventilation (Figure 1C,D) to spontaneous breathing (Figure 1E,F), the dead space grows to include parts of the inspiratory and expiratory tubes. We may resolve this by increasing the fresh gas flow,^6^ using pressure to support ventilation,^2^ and even using mechanical ventilation. Nonetheless, increasing fresh gas flow does not only work ineffectively; but it also wastes anesthetic gas and contributes to pollution.^6^ Pressure support ventilation and mechanical ventilation can cause elevated pressure,^7^ pulmonary atelectasis,^8^ and present challenges in the recovery process.^9^ Indeed, one‐way valves could directly reduce the dead space.^10^ But traditional one‐way valves added weight and ventilation resistance to the breathing tubing, leading to its abandonment.^10^ The feasibility of optimizing the design of one‐way valves to decrease their weight and ventilation resistance to the breathing tubing remains uncertain.

**FIGURE 2:**
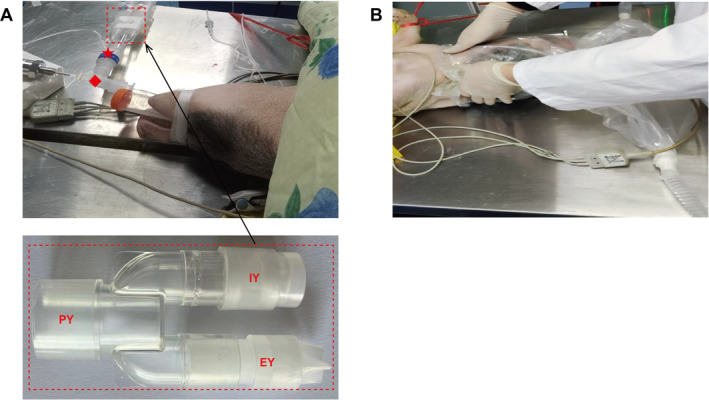
Inhalation anesthesia of the piglets in the circle breathing system. (A) Working state of the Y‐piece with one‐way valves. ★ indicates the location of monitoring end‐tidal carbon dioxide in the breathing tubing. ◆ indicates the location of monitoring airway pressure in the breathing tubing. (B) Inhalation induction with the induction bag. EY, expiratory Y‐piece; IY, inspiratory Y‐piece; PY, patient Y‐piece.

To address the above issues, we designed anti‐rebreathing tubing containing two one‐way valves with opposite openings in the inspiratory and expiratory Y‐pieces (Figure 2A). Theoretically, it prevents CO_2_ rebreathing from inspiratory and expiratory tubes during spontaneous breathing (Figure 1G,H) and increases resistance to breathing. This study tested the hypothesis that one‐way valves inside the breathing tubing in the circle breathing system can reduce dead space during spontaneous breathing and that the benefits to the lungs outweigh the risks associated with them.

## 2 MATERIALS AND METHODS

### 2.1 Animals and ethics

Six healthy female piglets (*Sus scrofa domestica* and *Bama miniature pigs*) aged 68–71 days were included. The pig is a commonly used animal model to simulate human breathing.^11^ Prior to the experiment, each piglet was fasted for 6 h and denied water for 2 h in a separate cage from their usual feeding setting. This animal study was performed at the Chongqing Academy of Animal Sciences, Chongqing, China, after authorization from its Animal Ethics Committee (File number: Cqaa2022006).

### 2.2 Breathing tubing

Two types of breathing tubing were used: (1) Anti‐rebreathing tubing containing one‐way valves with opposing openings in the inspiratory and expiratory Y‐pieces (Figure 2A) of 1.2 m standard breathing tubing for children (the mean diameter is 15 mm). We made the one‐way valve out of silicone, which was negligible in weight. The silicone had a Shore A hardness of about 20 A. A patent details the structure of a one‐way valve.^12^ (2) Traditional tubing was identical to anti‐rebreathing tubing, except for one‐way valves.

### 2.3 Sevoflurane induction

This experiment used only sevoflurane. The piglet's head and neck were inserted into an induction bag (Figure 2B). Two tubes at the bottom of the induction bag were connected to the inspiratory and expiratory ports of Fabius GS Premium (Dräger Medical, Lübeck, Germany). We kept the adjustable pressure‐limiting valve at 0 cm H_2_O throughout the experiment. Sevoflurane (Hengrui, Shanghai, China) was started at 8% in 6 L/min of pure oxygen and reduced to 3% for 15 min after the piglet stopped struggling. A cuffed endotracheal tube (internal diameter 5.0 mm) was placed. Neither pressure support ventilation nor mechanical ventilation.

### 2.4 Randomization and blinding

After induction, the piglets were randomly divided into two groups of three. The piglets breathed spontaneously, the traditional group via the traditional tubing, and the anti‐rebreathing group via the anti‐rebreathing tubing. All breathing tubing had opaque white tape on the Y‐pieces, and the one‐way valves were translucent and white (Figure 2A). The computer‐generated randomization group numbers were sealed in envelopes. All personnel, except for the anesthesiologist, were unaware of the piglet's group assignment.

### 2.5 Spontaneous breathing

Piglets breathed spontaneously for 4 h. Four hours is sufficient and necessary for the time‐consuming magnetic resonance imaging (MRI), such as multiple body parts.^13^ It is also suitable for surgeries that can be performed with light sedation and nerve block, such as proximal humerus fracture operations.^14^

Oxygen saturation was measured through the ear by a finger clip sensor. Piglets were kept warm by measuring their body temperature through the anus. End‐tidal carbon dioxide (ETCO_2_) and airway pressure (Paw) were monitored between the endotracheal tube and patient Y‐piece (Figure 2A). The anesthesia workstation continuously measured ETCO_2_ and TV for five breaths per hour and averaged the results. A pressure sensor (Saiennuo, Wuxi, China) measured the airway pressure. Paw was Paw range (the difference between the means of the highest and lowest 10 Paw readings in 1 min) was calculated. Paw's positive pressure during mechanical ventilation has correlated favorably with the degree of ventilator‐induced lung injury,^15^ and Paw's negative pressure during forceful spontaneous breathing has caused self‐inflicted lung injury.^16^ TV was divided by body weight to yield the TV ratio. Arterial blood gas was checked once per hour.

We found that 2.25% sevoflurane was a suitable sedating concentration for tracheal intubated piglets in preliminary experiments. Sevoflurane was started at 2.25% in 2 L/min with 30% oxygen and 70% nitrogen, and the concentration was increased by the lowest vaporizer setting when the piglet moved. Sevoflurane was decreased by the lowest vaporizer setting if heart rate dropped more than 15% after 0 h. Assisted ventilation (synchronized intermittent mandatory ventilation with pressure support mode: TV 6 mL/kg, respiratory rate 4 bpm, proportional pressure support 10 cm H_2_O) was started when ETCO_2_ was over 55 mmHg (severe hypercapnia)^17^ for more than 5 min and closed when it was below 55 mmHg for more than 5 min. The start‐close was counted as one instance. All parameters were determined in 5 min after assisted ventilation was turned off.

### 2.6 Animal sacrifice and lung tissue collection

The sedated piglet was sacrificed by bloodletting. The lungs were collected with minimal interference (Figure S1). Lung tissue was frozen at −80°C for mRNA sequencing and real‐time reverse transcription polymerase chain reaction (RT‐qPCR). Lung tissue was treated with 4% paraformaldehyde for immunohistochemistry. Lung tissue was treated with 10% neutral buffered formalin for histological analysis.

### 2.7 mRNA sequencing

Total RNA was isolated from the lung tissue using RNAiso Plus (Takara, Shiga, Japan), and the RNA purity was checked using a NanoPhotometer^®^ spectrophotometer (IMPLEN, Munich, Germany). mRNA was purified from total RNA using poly‐T oligo‐attached magnetic beads. mRNA sequencing libraries were generated using the NEBNext^®^ Ultra™ RNA Library Prep Kit for Illumina^®^ (NEB, Ipswich, USA) following the manufacturer's instructions, and index codes were added to attribute sequences to each sample. The original sequencing count data were normalized by the DESeq2 package,^18^ and the thresholds were set as fold‐change (FC) |logFC| > 0.585 and *p* < 0.05. Enrichment analysis was performed with Database for Annotation, Visualization and Integrated Discovery (DAVID) to identify the Gene Ontology categories and Kyoto Encyclopedia of Genes and Genomes (KEGG) pathways involving the differentially expressed genes, and the thresholds were set as *p* < 0.05 and count > 2.^19^ Cytoscape was used for building protein‐protein interaction (PPI) networks and topological data analysis, and the interaction score was set at ≥ 0.7.^20^

### 2.8 RT‐qPCR

Total RNA was isolated from the lung tissue using RNAiso Plus (Takara, Shiga, Japan). Complementary DNA was obtained using the PrimeScript™ RT reagent Kit with gDNA Eraser (Perfect Real Time) (Takara, Shiga, Japan) following the manufacturer's instructions. Relative gene expression was measured by real‐time PCR using PerfectStart^®^ Green qPCR SuperMix (TransGen, Beijing, China). Gene expression was quantified using the 2^−ΔΔCt^ method and normalized to glyceraldehyde‐3‐phosphate dehydrogenase, with the traditional group serving as the control. The target genes were identified via mRNA sequencing. The primer sequences are listed in supporting information (Table S1).

### 2.9 Immunohistochemistry and histology

Paraffin‐embedded lung tissue was cut into 4‐μm‐thick sections. Paraffin sections were stained with phosphorylated histone 2AX (γ‐H2AX) antibody (Bioss, Beijing, China) following the manufacturer's instructions for immunohistochemistry. The ImageJ immunohistochemistry profiler plugin was used to assess the immunohistochemistry optical density scores.^21^ Paraffin sections from each lobe (one dorsal and one ventral in the middle of the lobe) of the lungs were stained with hematoxylin and eosin for histology. Analysis and scoring were done by a pathologist who was blinded to the grouping of the piglets. The sections were given scores from 0 to 3 (0 = 0%–25%, 1 = 25%–50%, 2 = 50%–75%, and 3 = 75%–100%) for each of the following characteristics: interstitial edema, hemorrhage, leukocyte infiltration, atelectasis, hyaline membrane formation, and alveolar damage or rupture.^22^

### 2.10 Statistical analysis

Statistical analysis was performed using SPSS 27.0 (IBM), and graphs were drawn using Prism 9 (GraphPad). A student's *t* test was used to compare the two groups, with the parametric outcomes reported as the mean ± standard deviation or mean [95% confidence interval for mean]. For parameters with repeated measures, we used a linear mixed model adjusted with Bonferroni correction for multiple comparisons. The interaction between ETCO_2_ and arterial carbon dioxide tension (paCO_2_) was analyzed with Pearson's correlation analysis, and the relationship between Paw range and TV ratio was analyzed with Pearson's correlation analysis. Due to insufficient prior data, no a priori sample size calculations could be made to evaluate the effect of different breathing tubing on spontaneous breathing in the circle breathing system. The sample size for each group was set to be three based on the findings of our preliminary experiment, which indicated that piglets were unable to prevent hypercapnia in the circle breathing system over a 4‐h period of spontaneous breathing using traditional tubing. A two‐sided *p* < 0.05 was considered statistically significant.

## 3 RESULTS

### 3.1 Vital signs, respiratory characteristics, and arterial blood gas

The groups did not significantly differ in body weight (traditional group vs. anti‐rebreathing group: 15.2 [12.0; 18.3] vs. 13.9 [12.7; 15.1] kg, *p* = 0.182). The vital signs of the piglets changed similarly during spontaneous breathing (Table 1).

**TABLE 1:** Means [95% confidence interval for mean] or mean ± standard deviation of vital signs, respiratory characteristics, and arterial blood gas at 0 and 4 h during spontaneous breathing via two types of breathing tubing.

	Time = 0 h		Time = 4 h		Linear mixed model^a^	
	Trad (*N* = 3)	Anti (*N* = 3)	Trad (*N* = 3)	Anti (*N* = 3)	*F* Value	*p* Value
Vital signs						
Heart rate (bpm)	142.0 [116.4; 167.6]	161.0 [135.4; 186.6]	172.7 [153.3; 192.0]	165.0 [145.6; 184.4]	0.36	0.555
Temperature (°C)	36.3 [34.8; 37.8]	37.0 [35.5; 38.6]	38.1 [37.0; 39.1]	38.5 [37.5; 39.6]	0.30	0.591
SpO_2_ (%)^b^	97.7 ± 0.6	98.3 ± 1.5	98.3 ± 1.5	98.3 ± 1.2	0.22	0.647
Respiratory characteristics						
Respiratory rate (bpm)	31.3 [19.8; 42.9]	36.0 [24.5; 47.5]	32.7 [23.0; 42.4]	36.0 [26.3; 45.7]	1.67	0.213
TV (mL)	110.5 [92.7; 128.2]	106.4 [88.6; 124.2]	122.2 [98.6; 145.8]	120.1 [96.4; 143.7]	1.13	0.303
TV ratio (mL/kg)^c^	7.3 [6.0; 8.5]	7.7 [6.4; 8.9]	8.0 [6.7; 9.4]	8.6 [7.3; 10.0]	1.89	0.189
ETCO_2_ (mmHg)	51.1 [45.3; 56.8]	47.0 [41.2; 52.8]	43.0 [40.2; 45.8]	39.4 [36.6; 42.2]	12.53	0.003**
Paw range (cm H_2_O)^d^	7.7 [4.1; 11.4]	9.8 [6.1; 13.4]	7.4 [3.4; 11.4]	10.7 [6.7; 14.7]	14.88	0.001**
Sevoflurane (%)^e^	2.7 [1.8; 3.5]	2.8 [1.9; 3.6]	2.8 [2.3; 3.2]	2.5 [2.1; 2.9]	1.39	0.256
Arterial blood gas						
pH	7.36 [7.31; 7.42]	7.37 [7.31; 7.43]	7.44 [7.39; 7.49]	7.47 [7.43; 7.52]	5.89	0.031*
paCO_2_ (mmHg)	54.0 [51.9; 56.1]	52.3 [50.3; 54.4]	46.3 [42.6; 50.0]	40.7 [37.0; 44.4]	16.00	0.002**
paO_2_ (mmHg)	101.7 [82.4; 121.0]	98.0 [78.7; 117.3]	106.0 [92.5; 119.5]	99.7 [86.2; 113.1]	0.55	0.469
Glucose (mmol/L)	3.4 [1.4; 5.5]	3.7 [1.6; 5.8]	4.8 [0.9; 8.7]	6.4 [2.5; 10.3]	1.18	0.293
Lactate (mmol/L)^b^	2.0 ± 1.0	3.6 ± 1.6	0.9 ± 0.2	1.4 ± 1.4	2.23	0.165

Abbreviations: Anti, anti‐rebreathing group; ETCO_2_, end‐tidal carbon dioxide; PaCO_2_, arterial carbon dioxide tension; paO_2_, arterial oxygen tension; Paw, airway pressure; SpO_2_, oxygen saturation measured by pulse oximetry; Trad, traditional group; TV, tidal volume.

^a^ The repeated measurements between the two groups at five time points (0 h, 1 h, 2 h, 3 h, and 4 h) were analyzed using a linear mixed model.

^b^ When the value of the 95% confidence interval exceeds the permissible range of values, it is represented as the mean ± standard deviation.

^c^ The TV ratio was calculated as TV divided by body weight.

^d^ The Paw range was the difference between the means of the highest and lowest 10 Paw readings in 1 min.

^e^ The sevoflurane concentration was the setting value of the vaporizer.

The level of significance is expressed as * and ** for *p* < 0.05 and *p* < 0.01, respectively.

**FIGURE 3:**
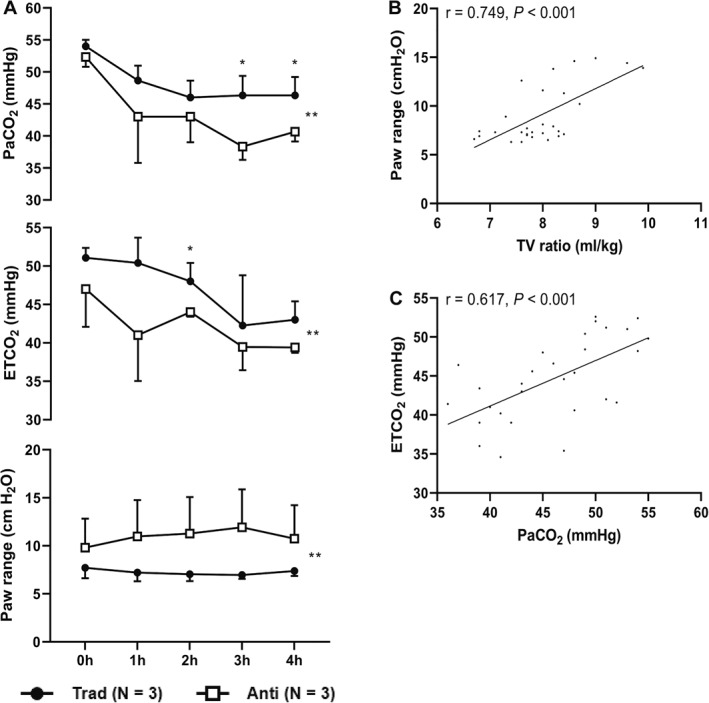
The main differences between the two groups and Pearson's correlation analysis. (A) The changes in carbon dioxide and resistance to breathing over time between the two groups. The Paw range was the difference between the means of the highest and lowest 10 Paw readings in 1 min. For parameters with repeated measures (0 h, 1 h, 2 h, 3 h, and 4 h), we used a linear mixed model and adjusted it with Bonferroni correction for multiple comparisons. Error bars represent the standard deviation. The level of significance is expressed as * and ** for *p* < 0.05 and *p* < 0.01, respectively. (B) Pearson's correlation plots of the relationship between TV ratio and Paw range in all piglets. The TV ratio was calculated as TV divided by body weight. (C) Pearson's correlation plots of the relationship between paCO_2_ and ETCO_2_ in all piglets. Anti, anti‐rebreathing group; ETCO_2,_ end‐tidal carbon dioxide; paCO_2_, arterial carbon dioxide tension; Paw, airway pressure; Trad, traditional group; TV, tidal volume.

During the spontaneous breathing, there were no significant differences in respiratory rate or TV between the two groups (Table 1). For 4 h, all three piglets with mild hypercapnia (45 mmHg < paCO_2_ ≤ 55 mmHg)^17^ spontaneously breathed via the traditional tubing experienced severe hypercapnia and required assisted ventilation. However, the other three piglets with mild hypercapnia developed normocapnia during spontaneous breathing via anti‐rebreathing tubing without assisted ventilation (3 [0.5; 5.5] times vs. none, *p* = 0.007) in less than 3 h and maintained proper paCO_2_ (Figure 3A). Over five time points (0 h, 1 h, 2 h, 3 h, and 4 h), PaCO_2_ dropped less in the tradition group than in the anti‐rebreathing group (Table 1, Figure 3A). At three and 4 hours, PaCO_2_ was higher in the traditional group than the anti‐rebreathing group (46.3 [42.1; 50.5] vs. 38.3 [34.1; 42.5] mmHg, *p* = 0.020; 46.3 [42.6; 50.0] vs. 40.7 [37.0; 44.4], *p* = 0.040). Over five time points (0 h, 1 h, 2 h, 3 h, and 4 h), the one‐way valves led to a higher Paw range in the anti‐rebreathing group (Table 1, Figure 3A). TV ratio was positively correlated with Paw range (*r* = 0.749, *P* < 0.001) (Figure 3B). paCO_2_ was positively correlated with ETCO_2_ (*r* = 0.617, *P* < 0.001) (Figure 3C).

### 3.2 mRNA‐based methods

**FIGURE 4:**
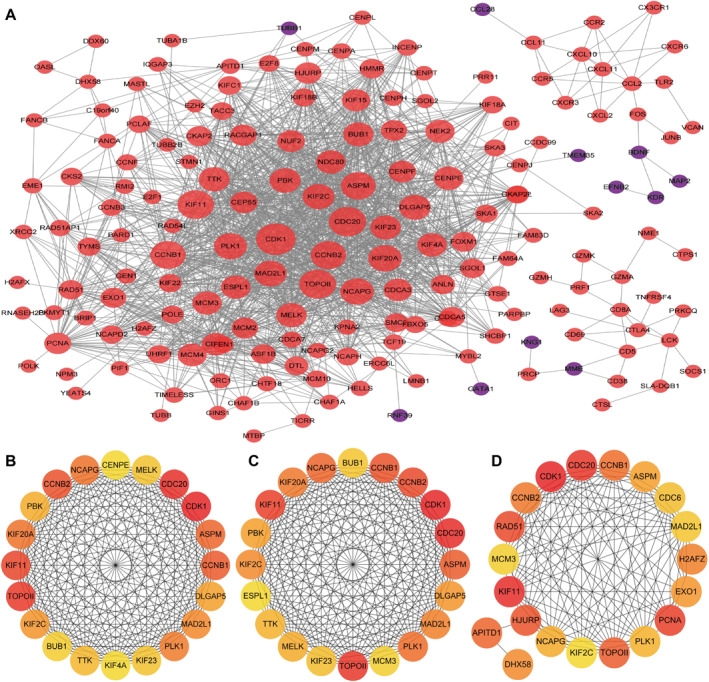
Protein‐protein interaction (PPI) networks of the proteins in piglets' lung tissue mRNA sequencing. (A) PPI network analysis of the 178 gene product proteins that were significantly regulated. The interaction score was set at ≥0.7. According to spontaneous breathing via traditional tubing versus anti‐rebreathing tubing, the red nodes represent upregulated gene product proteins, and the purple nodes represent downregulated gene product proteins. The larger the node, the stronger the connection. The top 20 gene product proteins according to the topology algorithms for degree (B), closeness (C), and betweenness (D). In (B, C, D), the darker the color, the higher the ranking.

**FIGURE 5:**
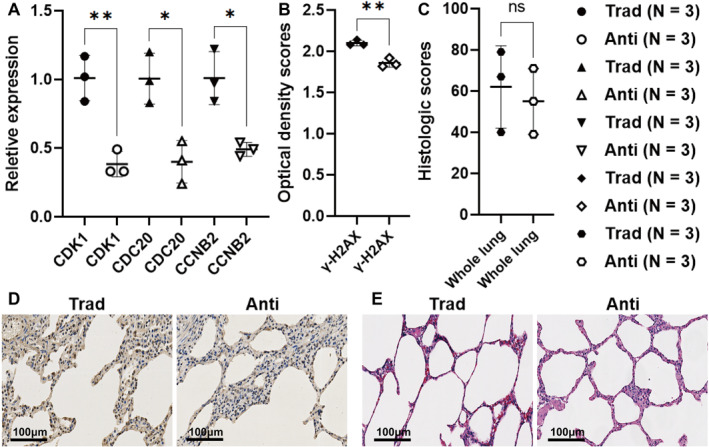
Lung tissue analysis after spontaneous breathing. (A) Relative expression of the genes in piglets' lung tissue was determined by RT‐qPCR. (B, D) Lung tissue stained with the *γ*‐H2AX antibody. (C, E) Lung tissue stained with hematoxylin and eosin (the histologic score is the total score of all lobes). Error bars represent the standard deviation. The level of significance is expressed as ns, *, and ** for no significance, *p* < 0.05, and *p* < 0.01, respectively. Anti, anti‐rebreathing group; CCNB2, cyclin B2; CDC20, cell division cycle 20; CDK1, cyclin‐dependent kinase 1; *γ*‐H2AX, phosphorylated histone 2AX; IHC, immunohistochemistry; RT‐qPCR, real‐time reverse transcription polymerase chain reaction; Trad, traditional group.

Lung tissue mRNA sequencing revealed 747 differentially expressed genes: 440 upregulated and 307 downregulated genes in the traditional group versus the anti‐rebreathing group. According to the methods part, a PPI network was made with 178 gene product proteins (Figure 4A). Eleven genes were found through mRNA sequencing by integrating and intersecting the top 20 genes from three topology algorithms (Figure 4B–D). According to the PPI networks (Figure 4) and KEGG pathway enrichment analysis (Figure S2), cyclin‐dependent kinase 1 (CDK1), cell division cycle 20 (CDC20), cyclin B2 (CCNB2), cyclin B1, polo‐like kinase 1, and mitotic arrest deficient 2 like 1 of these 11 genes were enriched in the cell cycle, DNA replication, and homologous recombination. RT‐qPCR, like mRNA sequencing, showed that CDK1, CDC20, and CCNB2 were more highly expressed in the lung tissue of the traditional group (Figure 5A).

### 3.3 Paraffin‐embedded lung tissue analysis

γ‐H2AX expression increased in the lung tissue of the traditional group (Figure 5B,D). Histologic scores of the whole lungs were not significantly different between the two groups (Figure 5C,E).

## 4 DISCUSSION

The circle breathing system, which is commonly used for gas mixture flows between the anesthesia workstation and patient, is not suitable for spontaneous breathing due to the dead space caused by the breathing tubing. Our study found that when piglets used the circle breathing system during spontaneous breathing, anti‐rebreathing tubing with one‐way valves in the Y‐piece discharged CO_2_ more effectively than traditional tubing. During 4 h of spontaneous breathing, piglets' paCO_2_ in the anti‐rebreathing group returned to normal (35 mmHg ≤ paCO_2_ ≤ 45 mmHg)^17^ without the need for assisted ventilation, whereas the traditional group could not. Furthermore, spontaneous breathing via the anti‐rebreathing tubing was beneficial rather than harmful to the lungs.

After induction, all piglets developed mild hypercapnia; only spontaneous breathing via the anti‐rebreathing tubing normalized paCO_2._ Because the CO_2_ absorption canister in the anesthesia workstation ensures the gas output is CO_2_‐free, the only source of CO_2_ in the breathing tubing is the piglet itself. All piglets in this study had the same basic conditions and sedative experience, and there were no significant differences in glucose, lactate, temperature, or heart rate between the groups, so they likely had the same metabolic rate. And all piglets had adequate arterial oxygen tension^23^ throughout the observation, so it is reasonable to conclude they produced the same amount of CO_2_. Moreover, TV and respiratory rate did not significantly differ between the groups, suggesting all piglets' spontaneous breathing movements had the same efficiency. Only piglets that spontaneously breathed via the traditional tubing developed severe hypercapnia, requiring occasional assisted ventilation. Although assisted ventilation clearly helped to eliminate CO_2_ in the body, our experiment still revealed a noteworthy lower PaCO_2_ in the anti‐rebreathing group. Therefore, the anti‐rebreathing tubing reduced dead space during spontaneous breathing compared to the traditional tubing, which should explain the difference in paCO_2_.

ETCO_2_ can be monitored in a noninvasive and continuous manner. While numerous studies have demonstrated the feasibility of assessing PaCO_2_ using ETCO_2_, they also highlight that ETCO_2_ often deviates from the actual CO_2_ level in the body due to various factors.^24^ That's exactly what we discovered in our study. Therefore, we used PaCO_2_ as the primary outcome and only used ETCO_2_ to determine whether the piglets required assisted ventilation.

Spontaneous breathing with sevoflurane sedation, because of the lack of intravenous access requirements and fast recovery, shows great potential for time‐consuming MRI and ambulatory surgery. The former can be achieved with a specific anesthesia workstation, such as the Fabius MRI (Dräger Medical, Lübeck, Germany), which can safely operate at high magnetic field strengths. Moreover, sevoflurane, an ideal anesthetic agent for the whole anesthesia procedure, activates spontaneous breathing by acting on the central nervous system.^25^ However, hypercapnia restricts spontaneous breathing in the circle system. Even mild hypercapnia can result in much deeper and quicker breathing,^3^ which can seriously disturb examinations or surgery. Our research addresses the problem that installing one‐way valves in breathing tubing can prevent hypercapnia during spontaneous breathing. Therefore, the circle breathing system could be suitable for spontaneous breathing with sevoflurane sedation via anti‐rebreathing tubing. This approach may take full advantage of inhalation anesthesia during spontaneous breathing, and the Fabius MRI would not just be compatible with mechanical ventilation under general anesthesia.

Although one‐way valves reduced PaCO_2_ in the body, which should be beneficial to the lungs, they also increased breathing resistance (a higher Paw range) during spontaneous breathing, which was harmful to the lungs. Therefore, research on lung tissue is necessary to allay the concerns about adding one‐way valves to the Y‐piece.^10^ The enrichment analysis of mRNA sequencing and RT‐qPCR showed that upregulated genes related to spontaneous breathing via traditional tubing were closely involved in genomic instability. This may result in *γ*‐H2AX‐identified double‐strand DNA breaks.^26^ Combined with higher *γ*‐H2AX expression in the lung of the traditional group, we suggested that spontaneous breathing via anti‐rebreathing tubing reduced lung damage in piglets by decreasing double‐strand DNA breaks through multiple mechanisms compared to traditional tubing. In particular, several studies have shown that permissive hypercapnia is beneficial just because of protective ventilation strategies such as low TV,^27^ and it is known that severe hypercapnia is an independent risk factor for mortality.^28^ We conclude that the anti‐rebreathing tubing increased resistance to breathing during spontaneous breathing, but not enough to cause noteworthy lung damage in 4 h; another possibility was that hypercapnia combined with occasional assisted ventilation caused more lung damage after the 4 h of spontaneous breathing via the traditional tubing.

In summary, breathing tubing with one‐way valves has the following advantages during spontaneous breathing over breathing tubing without one‐way valves in the circle breathing system: (1) reduces dead space; (2) causes fewer double‐strand DNA breaks in the lungs; (3) enhances the anesthesia advantages of the circle breathing system; and (4) makes sevoflurane sedation efficient and safe during spontaneous breathing.

### 4.1 Limitations

Some limitations of our study should be acknowledged. First, the study was limited to female piglets, and while we do not think that sex affected the results, it is still possible that there were some unforeseen effects. Second, all the piglets had mild hypercapnia after inhalation induction, so caution should be taken when applying our results to normocapnia. Third, animal CO_2_ generation was estimated only indirectly for technical reasons. Despite the fact that the factors that could affect the aerobic catabolism in piglets were homogenized as much as possible, there is still a possibility of the unexpected. Fourth, the piglets in the traditional group could not persistently breathe spontaneously because they required occasional assisted ventilation to treat severe hypercapnia. We minimized its impact by collecting all data 5 min after the assisted ventilation had stopped. Finally, because the mRNA sequencing results did not include typical cytokines related to ventilator‐associated lung injury, we did not study them, although we may do so in future investigations.

## 5 CONCLUSIONS

It may be feasible and safe for the sevoflurane‐sedative human to breathe spontaneously via the anti‐rebreathing tubing with one‐way valves in the circle breathing system. Before conducting a prospective randomized controlled clinical trial, we need to verify the feasibility of the anti‐rebreathing tubing for mechanical ventilation when necessary.

## AUTHOR CONTRIBUTIONS

Conceptualization: Pan Li, Li Jiang. Data curation: Pan Li, Weiping Wang, Wen Gao, Yanling Tan, Yu Hu. Formal analysis: Pan Li, Weiping Wang. Investigation: Pan Li, Wen Gao, Yanling Tan, Yu Hu. Methodology: Pan Li, Weiping Wang, Wen Gao. Resources: Pan Li, Li Jiang. Supervision: Li Jiang. Validation: Pan Li. Visualization: Pan Li. Writing – original draft: Pan Li. Writing – review & editing: Pan Li, Li Jiang. All authors read and approved the final manuscript.

## CONFLICT OF INTEREST STATEMENT

Pan Li holds a patent on *a one‐way valve and its related breathing tubing in the breathing system*. Weiping Wang, Wen Gao, Yanling Tan, Yu Hu, and Li Jiang have no conflicts of interest.

## ETHICS STATEMENT

The animal phase of this study was performed at the Chongqing Academy of Animal Sciences, Chongqing, China, after authorization from its Animal Ethics Committee (Chongqing, China; File number: Cqaa2022006).

## Supporting information

Supporting Information S1

## Data Availability

The datasets used and/or analyzed during the current study are available from the corresponding author on reasonable request. The mRNA sequencing data raw reads are available from the GenBank database (under accession number: PRJNA995196).
