# Supplementary material for: One‐way valves in breathing tubing reduce dead space during spontaneous breathing in anesthetized piglets
Source: Pediatr Discov. 2024 Aug 4;2(3):e2502. doi: 10.1002/pdi3.2502 (PMC12118172; doi:10.1002/pdi3.2502)
Supplement: Supplementary file 1 — Supporting Information S1 [file PDI3-2-e2502-s001.docx]

**Supporting Table 1.** The used primer sequences list

| **Primer** | **Sequence（5’-3’）** |
| --- | --- |
| GAPDH-F | AAGGTCGGAGTGAACGGATTT |
| GAPDH-R | CTCGCTCCTGGAAGATGGTG |
| CDK1-F | GGGGTCAGCTCGCTACTCAA |
| CDK1-R | TGCTAGGCTTCCTGGTTTCC |
| CDC20-F | AGTGGGCTCCCTCTGTTGG |
| CDC20-R | TTGGTGCTGCGTGAATGTCT |
| CCNB2-F | CCCGACGGTGTCTACTGATTT |
| CCNB2-R | GGCTTGTTGGCATTTGTTGTT |
| CCNB1-F | ACCAAATCAGGCAGATGGAAAT |
| CCNB1-R | GATGCTCTCCGAAGAAAATGC |
| PLK1-F | GGCTGCGTGCAGATCAACT |
| PLK1-R | GGAGGCTCAGGCGGTATGT |
| MAD2L1-F | GAGTTCTTCTCGTTTGGCATCA |
| MAD2L1-R | CATCTTTCAAGGACCTCACCACT |

Notes: CCNB1, cyclin B1; CCNB2, cyclin B2; CDC20, cell division cycle 20; CDK1, cyclin-dependent kinase 1; GAPDH, glyceraldehyde-3-phosphate dehydrogenase; MAD2L1, mitotic arrest deficient 2-like 1; PLK1, polo-like kinase 1.

**Figure Legends**

**Supporting Figure 1.** Process of collecting the whole lungs with minimal interference.

**Supporting Figure 2.** The top 10 terms associated with genes differentially expressed in piglets’ lung tissue after spontaneous breathing via two types of breathing tubing in the circle breathing system according to Gene Ontology (GO) and Kyoto Encyclopedia of Genes and Genomes (KEGG) enrichment. Genes were differentially expressed if they had |logFC| > 0.585 and *P* < 0.05. Pathways were ranked according to the *P* value. BP, biological process; CC, cellular component; FC, fold-change; GO, Gene Ontology; KEGG, Kyoto Encyclopedia of Genes and Genomes; MF, molecular function.

**Supporting Figure 1**


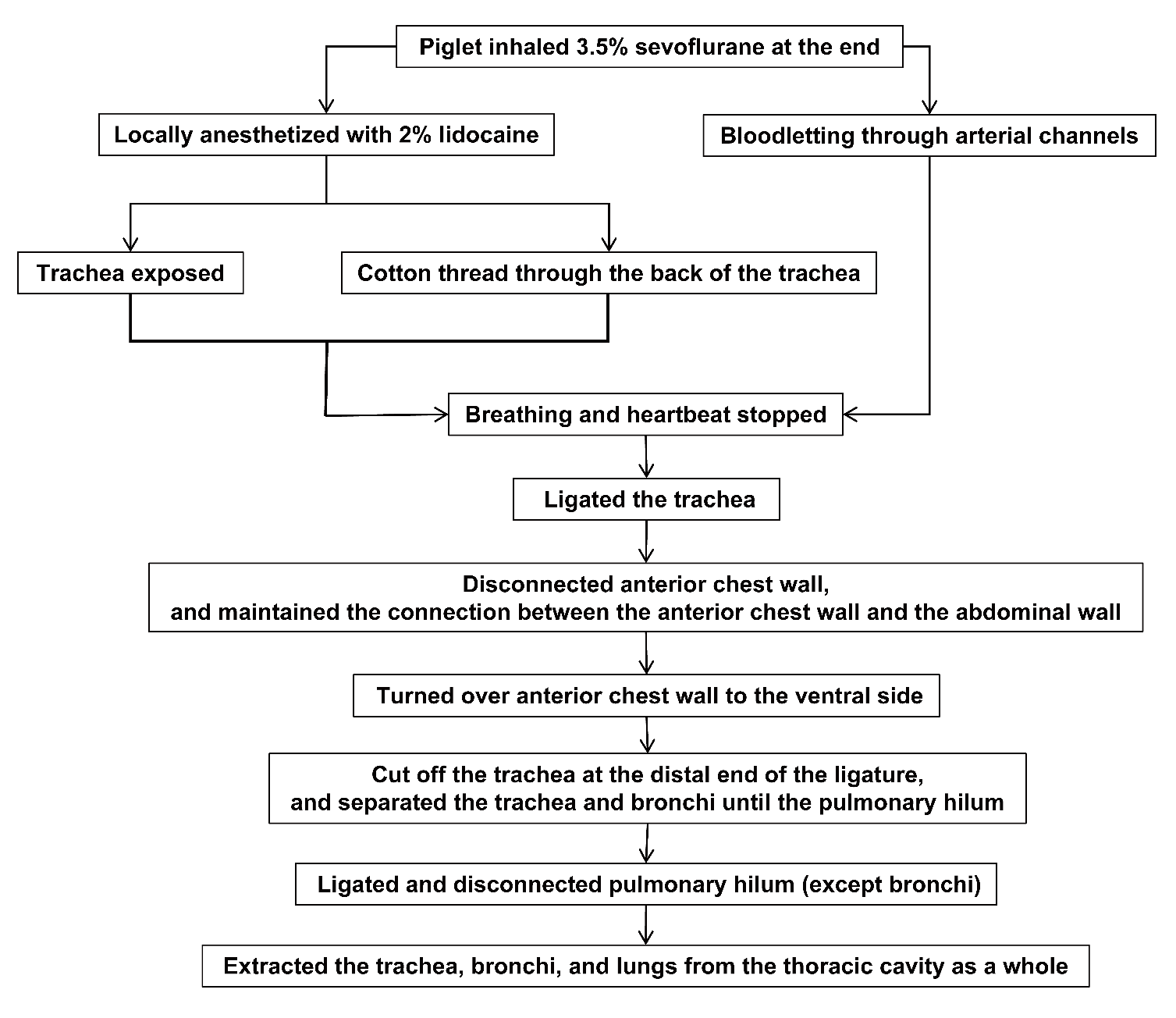


**Supporting Figure 2**

**
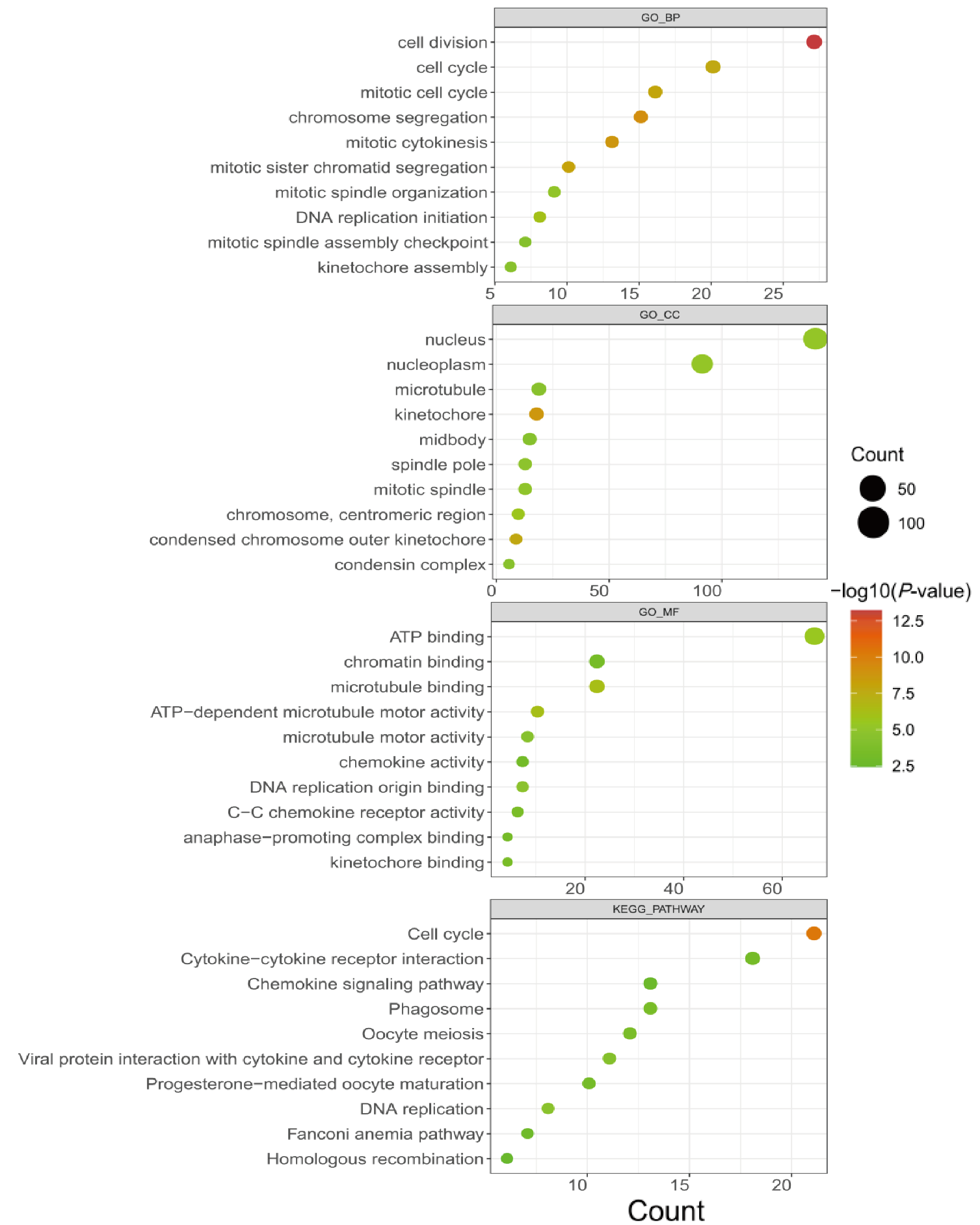
**
